# Trichinæ

**Published:** 1883-07

**Authors:** 


### TRICHINÆ.

This word—the plural of trichina—has its accent on the second syllable. It is from a Greek word meaning *hair*, and is the same of the hair-like worms sometimes found in the human muscles. The word "spiralis" is generally attached to it, and refers to the manner in which the parasite lies curled up in his tiny capsule.

When full grown, it would take eighteen of the males, placed end to end, to make an inch. The disease to which they give rise—at first often mistaken for muscular rheumatism—is called trichinosis, sometimes trichinaisis.

It was not until 1835 that the parasite was found in man. During the next twenty-five years it was proved that there was a connection between the disease in man and that of a hog; and in 1867 the parasite was found in the muscles of the latter. Whence the hog has derived it is an unsettled question.

As long as the hog lives the parasite remains dormant in the animal, like the chrysalis of the butterfly. But when the hog's flesh is eaten, the tiny capsules then are dissolved by the digestive juices, and trichinæ are set free.

A single meal may introduce many thousands of them—over a million, says one writer—into the stomach. Thus introduced they live from five to six weeks in the intestines, each one producing meanwhile a brood of at least one thousand five hundred. The latter soon migrate toward the muscles, following the course of the blood-vessels and nerves, and reaching their goal about the tenth day.

Here, in five or six months, they pass into a sort of chrysalis condition, to be freed from it only by the gastric juices of some other being. Similar migrations may follow, wave after wave. More or less, however, are swept out of the intestines, possibly to find their way back to their ancestral home in the swine.

The trichinæ have been found in every land. They have also been detected in the cat, dog, rabbit, rat, mouse, marmot, the wild hog of Europe, and even in the hippopotamus.

---

A FAVORITE antidote for rattlesnake poison in Mexico, is a strong solution of iodine in potassium iodide. Mr. H. H. Croft has tested some of the poison itself with this solution, and finds that a light-brown amorphous precipitate is formed, the insolubility of which explains the beneficial action of the antidote. When iodine cannot be readily obtained, a solution of potassium iodide, to which a few drops of ferric chloride has been added, can, perhaps, be used as an antidote to the poison.

